# Negotiating Chains: An Autoethnography from the Edge of Psychiatry and Anthropology

**DOI:** 10.1192/j.eurpsy.2026.11462

**Published:** 2026-07-31

**Authors:** V. S. Balilla

**Affiliations:** Advanced Comprehensive Center for Mental Health, Mariveles Mental Wellness and General Hospital, Mariveles, Bataan, Philippines

## Abstract

**Introduction:**

Shackling of individuals with mental illness remains a significant human rights issue in the Philippines, deeply embedded within complex sociocultural and systemic contexts. This practice, often a hidden last resort for families, represents a critical failure in the continuum of care.

**Objectives:**

This study aims to investigate the author’s confrontation with shackling during Psychiatry and Public Mental Health residency. It seeks to: (1) examine the negotiation of ethical dilemmas and clinical realities from the liminal space between psychiatry and anthropology; and (2) identify factors perpetuating the practice to inform pathways toward more humane care.

**Methods:**

The study employs autoethnography, drawing on personal narrative, reflective practice, and clinical interactions at Mariveles Mental Wellness and General Hospital. A dual lens of human rights frameworks and social constructionism informs the critical analysis of the author’s experiences and provider viewpoints.

**Results:**

The analysis reveals profound emotional and ethical dilemmas for the clinician, caught between anthropological understandings of familial desperation and psychiatric mandates for safety and intervention. Systemic failures—including poverty, stigma, insufficient community mental health services, and policy gaps—were identified as primary drivers compelling families to use chains. The author’s dual disciplinary position highlighted both the tensions and the potential synergy between psychiatric and anthropological perspectives.

**Conclusions:**

Shackling is a multifaceted problem rooted in systemic inadequacies and sociocultural determinants, demanding responses that transcend purely clinical interventions. Integrating psychiatric practice with anthropological insight is crucial for developing rights-based, culturally sensitive solutions. Upholding human dignity requires systemic reform, sustained community and family support, and critical self-reflection among healthcare providers.

**Disclosure of Interest:**

None Declared

